# The shape of chiropractic in Europe: a cross sectional survey of chiropractor’s beliefs and practice

**DOI:** 10.1186/s12998-019-0237-z

**Published:** 2019-04-10

**Authors:** Halldór Fannar Gíslason, Jari Kullervo Salminen, Linn Sandhaugen, Andreas Stenseth Storbråten, Renske Versloot, Inger Roug, Dave Newell

**Affiliations:** 1 Reykjavik, Iceland; 2 Tampere, Finland; 3 Oslo, Norway; 4 Wijchen, The Netherlands; 5 AECC University College, Parkwood Road, Bournemouth, England, UK

**Keywords:** Orthodox, Non-orthodox, Chiropractic, Practice, Beliefs, Paradigms

## Abstract

**Background:**

The chiropractic profession both in the past and presently has diverse opinions concerning different health care approaches and the science or otherwise that underpins them. Previous research has reported that adherence to unorthodox descriptions of chiropractic were associated with types of practice behavior considered outside of acceptable evidence-based guidelines in Canada. However, this type of investigation has not been repeated in a European context and such relationships may be different.

**Methods:**

A survey was disseminated amongst European chiropractors during early 2017. Dissemination was through an on-line platform with links to the survey being sent to all European chiropractic associations regardless of European Chiropractors’ Union (ECU) membership and additionally through the European Academy of Chiropractic (EAC). Social media via Facebook groups was also used to disseminate links to the survey.

**Results:**

One thousand three hundred twenty and two responses from chiropractors across Europe representing approximately 17.2% of the profession were collected. Five initial self-determined chiropractic identities were collapsed into 2 groups categorised as orthodox (79.9%) and unorthodox (20.1%). Analysis of responses to a range of questions stratified by such groups was carried out. When comparing the percentage of new patients chiropractors x-rayed, 23% of the unorthodox group x-rayed > 50% of their new patients compared to 5% in the orthodox group. Furthermore, the proportion of respondents reporting > 150 patient encounters per week in the unorthodox group were double compared to the orthodox (22 v 11%). Lastly the proportion of those respondents disagreeing or strongly disagreeing with the statement *“In general, vaccinations have had a positive effect on global public health”* was 57 and 4% in unorthodox and orthodox categories respectively. Logistic regression models identified male gender, seeing more than 150 patients per week, no routine differential diagnosis, and not strongly agreeing that vaccines have generally had a positive impact on health as highly predictive of unorthodox categorisation.

**Conclusions:**

Despite limitations with generalisability in this survey, the proportion of respondents adhering to the different belief categories are remarkably similar to other studies exploring this phenomenon. In addition, and in parallel with other research, this survey suggests that key practice characteristics in contravention of national radiation guidelines or opposition to evidence based public health policy are significantly more associated with non-orthodox chiropractic paradigms.

## Background

The history of the chiropractic profession is one characterised by a trajectory from early theories that attempted to explain the improvements seen during chiropractic care to increasingly embracing modern science, research methodology and evidence.

However, the profession has not made this journey in unison. The present state might reasonably be characterised as a spectrum of ideas within which two broad and polarised explanatory frameworks exist to explain positive outcomes seen in practice [1]. The degree of adherence to these paradigms varies across chiropractic institutions and geographical jurisdictions and has been studied previously [2–4]. One paradigm is characterised by a broad group of descriptions as to how chiropractic care generates patient outcomes seen in practice. These range from a musculoskeletal and structurally focused explanation [5], more neurological emphases [6] to broad ideas around prevention and wellness. Whilst it is unlikely that any of these explanations are uniquely true descriptions of the mechanisms by which patients improve they either have some evidential support or at the very least provide biologically plausible explanations. An alternative group of paradigms hold more strongly to historical ideas and theories espoused at the inception of the profession. A proportion of the profession still consider these theories and explanatory frameworks to be relevant either as described by the original unabridged teachings of the Palmers or merged together with a mix of scientific, pseudo-scientific and philosophical elements that is described in some quarters as constituting a new healthcare paradigm. To some degree or another this unorthodox explanatory framework has its roots in vitalism [7] and is centred on the body’s ability to heal itself if theorised barriers to such healing are removed. Amongst legitimate ideas within this paradigm are those such as addressed in mainstream public health initiatives including healthy eating and increased activity. However, this paradigm is also associated with less evidenced impediments to health such as spinal neuro-biomechanical lesions described as subluxations [8, 9].

Strong vitalistic versions of this explanatory framework propose a life force (Innate Intelligence) that is theorised to be carried by the nervous system [7]. This belief includes the idea that manipulating the spine to remove restrictions or ‘chiropractic subluxations’ can restore health and some chiropractors see themselves as the only individuals who can identify and remove such restrictions through unique ‘chiropractic manipulations’ and restoring the full expression of such a life force. Despite a fundamental lack of supporting scientific evidence for this position, subgroups within the chiropractic profession continue to adhere to this or similar explanatory frameworks [2, 4].

A recent survey of Canadian chiropractors reported that around 18% of Canadian chiropractors chose such a vitalistic description to characterise what they do [2] and that such self-categorisation was also associated with unorthodox practices. Furthermore, a follow up study confirmed that such beliefs and ideologies were associated with a small subsection of US based chiropractic educational programs [4].

This study aimed to repeat such a survey within Europe as no data exists regarding such categorisation of beliefs or potential association with unorthodox practice.

## Methods

### Design and piloting

The survey instrument used in this study was a modified version of the original questionnaire [2]. Following a face validity study conducted amongst AECC University College faculty the structure of the questionnaire was altered in response to the majority of participants indicating a number of questions with the same emphasis. Specifically, an alteration made was regarding the original option [2] *“I treat a combination of general problems and biomechanical conditions”* (Biomechanical/General problems) which was removed due to similarities to *“I treat musculoskeletal or neuromusculoskeletal problems and may include specific disorders such as but not limited to low back and neck-related pain”* (Biomechanical) thus creating 5 chiropractic descriptions from the original 6.

### Dissemination

The survey was administered via Survey Monkey and distributed via consenting European national chiropractic associations. In addition, survey links were distributed extensively within a range of chiropractic Facebook groups. In addition, the European Academy of Chiropractic also assisted with distribution to its European members. Three reminders were sent to associations who didn’t confirm participation. All associations and the EAC were sent 2 e-mails to remind members to complete the survey between January 2017 and March 2017. One re-post of the survey in FB groups was also carried out to encourage participation.

### Target population

All chiropractors that were practicing in Europe at the time of the survey and capable of understanding and interpreting the English language were allowed to participate in this study. All chiropractors practicing outside of Europe were excluded from participation. This was achieved during data cleaning noting the ‘country’ question response in the survey. No compensation was offered to the participants. Because access to the entire population through a single e-mail list was not available sample randomisation was not possible. An introduction text at the beginning of the survey included information about confidentiality. *“Will my taking part in the study be kept confidential? Yes, we will anonymize all data we collect and we will not include any person identifiably information in the write-up of the study”.*

### Orthodox/unorthodox subgrouping

Orthodox and unorthodox categories were determined through a set of key questions (Table 1) within the survey with categories 1, 2, 3 and 4 defined a priori as orthodox and 5 as unorthodox as in the original study [2].

**Table 1:** Which ONE of the following best describes the predominant view you have of the conditions you treat?

1. I treat musculoskeletal and neuromusculoskeletal problems and include specific disorders such as but not limited low back and neck related pain.	
2. I treat the broadest spectrum of health concerns and may include lifestyle and wellness issues.	
3. I treat vertebral subluxation as a somatic joint dysfunction and/or related to functional or musculoskeletal problems.	
4. I treat a combination of biomechanical and organic/visceral complaints.	
5. I treat vertebral subluxation as an encumbrance to the expression of health - vertebral subluxation is seen as an entity in and of itself, which is corrected to benefit patient well-being.	

### Statistical analysis

Descriptive analysis was used to describe the respondents’ characteristics and to determine any differences in responses between orthodox and unorthodox categories. In addition, a forward binary logistic regression model was used to model factors within the survey instrument that predicted membership of the unorthodox category. Countries with less than a hundred chiropractors according to the latest European Chiropractors’ Union (ECU) figures (personal communication, 12 June 2017) and countries with a response rate less than 10% were excluded from the country analysis.

## Results

All European associations, both members of the ECU and non member associations (*n* = 37) were approached. Of these, e-mails were received from 15 associations confirming participation. Only one association specifically declined to participate.

Thirteen hundred and twenty-two (*N* = 1322) chiropractors returned completed questionnaires. This constituted a 17.2% return rate based on estimated figures of 7680 chiropractors in 2017 (personal communication, 12 June 2017).

Table 2 shows the general demographic characteristics of the respondents indicating a skewing toward male practitioners at the level of survey respondents. Most respondents entered chiropractic training from secondary school. However, over 1/3 entered with a University degree. The largest age group were 30 to 39-year olds and the average career length was around 14 years. Less than 1% of the sample possessed a PhD.

**Table 2:** Characteristics of respondents (*N* = 1322)

Variables	N (%)	Mean (SD)
Gender (M)	835 (63.2)	–
Previous degree		
Secondary school	553 (42.1)	–
University Degree	461 (35.1)	–
Master’s Degree	94 (7.2)	–
PhD	12 (0.9)	–
Other	124 (9.5)	–
Age		
20–29	200 (15.4)	–
30–39	406 (31.2)	–
40–49	361 (27.7)	–
50–59	225 (17.3)	–
60–69	90 (6.9)	–
> 70	19 (1.5)	–
Years in practice	–	14.4 (10.8)

Returns were received from 23 European countries (Table 3). However, the return rates varied markedly with some countries with very few chiropractors such as Estonia and Portugal with high return rates while others with larger numbers such as Denmark and France returning very few.

**Table 3:** Return rate per country

Country	Respondents (N)	Total population^*^	Return rate (%)
Austria	1	2	50.0
**Belgium**	**62**	**120**	**51.7**
Cyprus	1	12	8.3
Denmark	41	879	4.7
Estonia	4	5	80.0
Finland	8	75	10.7
France	12	400	3.0
**Germany**	**81**	**179**	**45.3**
Greece	2	30	6.7
Hungary	5	6	83.3
Iceland	6	17	35.3
**Ireland**	**46**	**130**	**35.4**
**Italy**	**44**	**400**	**11.0**
Liechtenstein	2	5	40.0
Malta	1	7	14.3
**Norway**	**151**	**800**	**18.9**
Portugal	15	22	68.2
**Spain**	**90**	**330**	**27.3**
**Sweden**	**139**	**800**	**17.4**
**Switzerland**	**119**	**269**	**44.2**
**The Netherlands**	**109**	**400**	**27.3**
Turkey	3	7	42.9
**UK**	**330**	**3195**	**10.3**

*= personal communication ECU Data 2017, Bold= > 100 chiropractors in country and > 10% return rate

Categories that described the chiropractors’ views as to their practice and paradigm were a key element of this survey (Table 1) with Table 4 describing the proportions aligned with each description. The majority of chiropractors aligned with an MSK description of their practice (54%) with a further 20% describing their practice as addressing broad spectrum of health concerns including wellness or viscerosomatic issues. Around 27% included subluxation as part their practice description. However, 7% of these see the subluxation as a biomechanical lesion related to the MSK system leaving 20% describing such subluxations in a vitalistic paradigm that sees these restrictions as an encumbrance to health in and of themselves. Given that this last category is unsupported within a scientific or evidence-based paradigm it was defined as unorthodox with the remaining descriptions defined as orthodox given the varying degrees of supportive evidence or biological plausibility [2]. Table 5 indicates the proportions of orthodox and unorthodox self-selection within each of the countries that passed the criteria of having at least 100 practicing chiropractors and who achieved a greater than 10% return rate. In this data the highest proportion of unorthodox categorisation was found in Spain with Italy and Germany ranking 2nd and 3rd respectively. A range of countries were characterised by unorthodox proportions around 15–20% with the lowest rates being found in Switzerland, Belgium and Norway respectively.

**Table 4:** Self-selected chiropractic subgroups

Chiropractic subgroup	*N* (%)	Dichotomous grouping
General problems	163 (14.0)	Orthodox
Biomechanical	628 (54.0)	Orthodox
Biomechanical/Organic-Visceral	57 (4.9)	Orthodox
Subluxation as a somatic dysfunction	81 (7.0)	Orthodox
Subluxation as an obstruction to human health	233 (20.1)	Unorthodox

**Table 5:** Orthodox and unorthodox proportions in countries where > 100 chiropractors and > 10% return rate was achieved

Country	*N* (%) Orthodox	*N* (%) Unorthodox
Belgium	51 (92.7)	4 (7.3)
Germany^a^	43 (66.2)	22 (33.8)
Ireland	31 (79.5)	8 (20.5)
Italy	23 (59.0)	16 (41.0)
Norway	132 (93.0)	10 (7.0)
Spain	34 (43.6)	44 (56.4)
Sweden	101 (82.8)	21 (17.2)
Switzerland	102 (90.3)	11 (9.7)
The Netherlands	81 (82.7)	17 (17.3)
UK	236 (80.0)	59 (20.0)

^a^54% attended weekend course

Tables 6 and 7 describe a univariate logistic regression analysis reporting significant variables associated with orthodox/unorthodox categories. In terms of respondent characteristics, being male and between 30 and 39 years of age doubled the odds of belonging to the unorthodox grouping whereas only practicing in one country reduced the risk of belonging to this group (i.e. practicing in more than one country increased the risk of unorthodox self-categorisation). In terms of practice characteristics, reporting more than 100 patient visits weekly, not performing differential diagnosis and providing routine x-rays for more than 20% of new patients predicted membership of the unorthodox group. Beliefs that include the idea that chiropractic intervention effects the cause of conditions such as colic, enuresis, otitis media and dysmenorrhea were also significantly associated with unorthodoxy. In terms of public health issues such as vaccination; referring patients to a website, suggesting patients do their own research, providing pros and cons or providing information against vaccination, all predicted increased likelihood of belonging to the unorthodox group compared to an evidenced based approach of referring patients for advice to appropriately qualified health care professionals. Additionally, anything other than strongly agreeing that vaccines have had a positive effect on public health also significantly increased odds of belonging to the unorthodox grouping.

**Table 6:** Significant univariate practitioner and practice variables associated with unorthodox category

	*N*	OR (95% CI)	p
Practitioner Characteristics			
Female	418	1.0	–
**Male**	**738**	**2.1 (1.5 to 3.0)**	**0.00**
Age			
20–29	170	1.0	–
**30–39**	**372**	**1.6 (1.1 to 2.6)**	**0.04**
40–49	316	1.3 (0.8 to 2.2)	0.24
50–59	203	1.1 (0.6 to 1.8)	0.82
60–69	80	1.0 (0.5 to 2.1)	0.94
> 70	–	–	–
Always practiced in single country			
No	296	1.0	
**Yes**	**865**	**0.6 (0.4 to 0.8)**	**0.00**
Practice Characteristics			
Country			
Norway^*^	142	1.0	
Belgium	55	1.0 (0.3 to 3.4)	0.90
**Germany**	**65**	**6.7 (3.0 to 15.4)**	**0.00**
**Ireland**	**39**	**3.4 (1.2 to 9.3)**	**0.02**
**Italy**	**39**	**9.2 (3.7 to 22.7)**	**0.00**
**Spain**	**78**	**17.1 (7.8 to 37.4)**	**0.00**
**Sweden**	**122**	**2.7 (1.2 to 6.1)**	**0.01**
Switzerland	113	1.4 (0.6 to 3.5)	0.45
**Netherlands**	**98**	**2.8 (1.2 to 6.3)**	**0.02**
**UK**	**296**	**3.3 (1.6 to 6.6)**	**0.00**
Weekly Patient visits			
0–50	372	1.0	
51–100	456	1.3 (0.9 to 2.0)	0.15
**101–150**	**163**	**2.2 (1.4 to 3.6)**	**0.00**
**151 to 200**	**61**	**7.0 (3.9 to 12.7)**	**0.00**
**201 to 250**	**23**	**9.4 (3.9 to 22.8)**	**0.00**
**251 to 300**	**19**	**38.7 (10.8 to 132)**	**0.00**
**> 300**	**17**	**10.4 (3.8 to 28.6)**	**0.00**
Perform differential diagnosis			
Yes	1028	1.0	
**No**	**132**	**11.8 (7.9 to 17.6)**	**0.00**
Proportion of new patients given X ray (%)			
0–5	587	1.0	
6–20	291	1.0 (0.7 to 1.4)	0.89
**21–50**	**115**	**1.7 (1.1 to 2.6)**	**0.04**
**51–80**	**40**	**3.8 (2.0 to 7.4)**	**0.00**
**81–100**	**54**	**8.8 (4.8 to 15.9)**	**0.00**

Significant variables in bold, * = Norway had lowest proportion of unorthodox respondents and was used as the comparator, - = too few numbers to calculate

**Table 7:** Significant beliefs associated with unorthodox category

	N	OR (95% CI)	p
Conditions for which treatment effects cause			
Dysmenorrhea			
No	951	1.0	
**Yes**	**211**	**1.5 (1.1 to 2.1)**	**0.03**
Enuresis			
No	955	1.0	
**Yes**	**207**	**1.7 (1.2 to 2.4)**	**0.00**
Colic			
No	907	1.0	
**Yes**	**255**	**1.5 (1.2 to 2.0)**	**0.00**
Otitis Media			
No	959	1.0	
**Yes**	**203**	**1.6 (1.1 to 2.3)**	**0.00**
Advice on vaccines			
Suggest talk to an MD/Nurse	363	1.0	
**Refer to website (e.g. WHO)**	**56**	**3.6 (1.5 to 8.9)**	**0.00**
Provide information in support ^Ω^	70	–	–
**Provide with pros and cons**	**349**	**5.9 (3.4 to 10.4)**	**0.00**
**Suggest do their own research**	**249**	**12.3 (7.0 to 21)**	**0.00**
**Provide information against**	**63**	**40.0 (19.6 to 83)**	**0.00**
Response to question			
In general, vaccinations have had a positive effect on global public health?			
Strongly agree	300	1.0	
**Agree**	**335**	**30 (1–224)**	**0.00**
**Neutral**	**266**	**91 (12 to 660)**	**0.00**
**Disagree**	**155**	**259 (35 to 1894)**	**0.00**
**Strongly disagree**	**92**	**618 (83 to 4616)**	**0.00**

Ω = zero cases in unorthodox category so unable to calculate, Significant variables in bold

Final regression models adjusting for all significant univariate variables were constructed without (Table 8) and with (Table 9) the country variable. Only in the regression model shown in Table 9 did we exclude countries with few chiropractors and/or participants (*see Statistical Analysis in Methods section*). Characteristics that predicted unorthodoxy in both models were being male, seeing more than 150 patients per week, no routine differential diagnosis, and not strongly agreeing that vaccines have generally had a positive impact on health. These models explained over 50% of the variance and provided robust discriminatory power as illustrated by a large area under the curve (AUC) value for both models.

**Table 8:** Overall regression model for belonging to unorthodox category (without Country)

Variables in the equation	OR (95% Confidence Interval)	p
Male	1.8 (1.1 to 2.8)	0.01
Weekly Patient number		
151–200	2.3 (1.0 to 5.0)	0.04
201–250	4.4 (1.2 to 15.9)	0.02
251–300	14.4 (1.9 to 109)	0.01
> 300	5.1 (1.2 to 21.8)	0.03
% of new patient’s x ray (81–100)	3.4 (1.5 to 7.5)	0.00
Perform differential diagnosis (No)	3.8 (2.2 to 6.5)	0.00
Treatment effects cause of Enuresis (Yes)	1.8 (1.1 to 2.9)	0.02
Vaccines Advice		
Suggest do their own research	2.1 (1.0 to 4.3)	0.04
Provide information against	2.5 (1.1 to 6.4)	0.03
Response to question		
In general, vaccinations have had a positive effect on global public health?		
Agree	18.6 (2.4 to 142)	0.00
Neutral	33.9 (4.4 to 261)	0.00
Disagree	82.5 (10.5 to 648)	0.00
Strongly Disagree	134 (16.3 to 1103)	0.00

Nagelkerke = 0.51

AUC = 0.89 (95% CI; 0.87 to 0.92)

**Table 9:** Overall regression model for belonging to unorthodox category (with Country)

Variables in the equation	OR (95% Confidence Interval)	p
Country (Italy)	3.6 (1.2 to 11.3)	0.02
Male	1.9 (1.2 to 3.1)	0.01
Weekly Patient number		
101–150	2.3 (1.1 to 4.5)	0.02
151–200	2.8 (1.0 to 6.8)	0.03
201–250	5.5 (1.3 to 22.7)	0.02
251–300	13.0 (1.6 to 105)	0.02
> 300	6.0 (1.2 to 32.5)	0.04
Perform differential diagnosis (No)	3.5 (1.9 to 6.3)	0.00
Treatment effects cause of Enuresis (Yes)	2.0 (1.1 to 3.3)	0.01
Response to question		
In general, vaccinations have had a positive effect on global public health?		
Agree	20.5 (2.7 to 155)	0.00
Neutral	54.1 (7.2 to 404)	0.00
Disagree	151 (20.0 to 1139)	0.00
Strongly Disagree	287 (36.0 to 2880)	0.00

Nagelkerke = 0.53

AUC = 0.89 (95% CI; 0.87 to 0.92)

## Discussion

Contemporary opportunities afforded the chiropractic profession based on increasing alignment of guidelines and the scientific literature supporting the use of conservative care in the management of MSK conditions have been articulated previously [10]. However, to grasp such opportunities ongoing adherence to scientifically unsupported explanatory frameworks such as vitalism and the putative subluxation as a single impediment to health is increasingly untenable [7, 8, 10]. Indeed, such ongoing resistance to aligning with the scientific literature has been seen by some [8, 11–13] as the central issue in impeding professional legitimisation, scientific and societal support and inclusion within the wider health care professional landscape. Whilst professional legitimacy is probably high on the agenda of the chiropractic profession as a whole, such aims are counterfactual to non-scientific explanatory frameworks which persistently underlie accusations of unorthodoxy amongst the professions detractors [14].

It is possible that such explanatory frameworks may be admissible as simple analogies at the patient level, particularly taking into account the impact of congruence of explanations with patient existing beliefs and expectations [15], the necessity of building therapeutic alliance [16, 17] and the likely impact of explanatory rationales [18] and contextual factors on outcomes [19]. However, it is unlikely that these non-orthodox explanatory frameworks have or will engender traction and/or confidence in the scientific and health professional landscape [20] particularly at interprofessional, political and funder levels. More importantly, where adherence to such paradigms is associated with practice-based behaviours that run counter to clinical guidelines, scientific evidence and a patient centred approach, particularly where fear is used to leverage compliance, arguments of the supposed benign if misguided nature of such beliefs as excused by some in the profession are untenable [21].

In this regard, the results of this study revealed a number of factors that significantly predict the alignment of a respondent in this survey with an unorthodox chiropractic paradigm. Of these factors, less favourable views regarding vaccination and increased frequency of the use of x-rays with new patients were also described by McGregor et al. [2]. Additionally, a weekly patient load higher than 150 patients and opting not to perform a differential diagnosis also predict unorthodoxy although it is impossible to differentiate repeated visits by the same patients from high volumes of new patients.

Unlike in this study, *‘not performing a differential diagnosis’* was not explored by McGregor et al. [2]. This expectation is common in a European chiropractic educational context where clinicians are trained to generate or consider a differential diagnosis to guide further action. In some versions of chiropractic unorthodoxy clinicians do not see themselves as identifying or treating a condition or symptom, merely removing subluxations that they find. The association of unorthodoxy and lack of differential diagnosis in this study may be speculatively connected to such thinking.

Although unorthodox categorisation was associated with higher use of x-rays, no difference was found between the orthodox and unorthodox categories with regards to clinical justification for taking x-rays and the conditions treated. However, the results clearly indicate that unorthodox chiropractors tend to use x-ray investigations on their patients more often than their orthodox counterparts. As to why this type of practise exists, more qualitative research may be illuminating. However, religiosity underpinning early rationales for the use of x-rays have been suggested to be associated with ongoing adherence to such approaches [22]. Alternatively lack of knowledge of guidelines may underlie such behaviour and it is possible that educational interventions may change this behaviour [23]. Lastly, it is important to note that in some areas where chiropractors practice, a significantly older demographic may exist and this may precipitate a more risk averse approach to care that includes greater frequency of imaging as a precautionary stance. However, it is unlikely that those falling into the unorthodox group were differentially located within such demographics compared to the orthodox group and therefore this factor would not explain the greater use of x-rays amongst the unorthodox group.

Vaccination is presently part of a major public health debate. Whilst most clinicians across healthcare acknowledge overwhelming evidence of the benefits versus risk of vaccination programs this is not universally the case in all professions. In chiropractic some groups in the profession are closely associated with key campaigns and figures in what has become known as the ‘antivax’ community [24], with some studies reporting low future commitment of uptake amongst chiropractors of vaccination [25].

The reasons behind this are unclear and the question remains as to why chiropractors hold strong opinions on vaccinations when it is not their area of expertise or scope of practice. Lack of support for vaccinations in this study was highly correlated with alignment to the ‘vitalistic’ paradigm as articulated by the unorthodox option in the survey. Such vaccination scepticism has been found previously in chiropractic students [26]. Perhaps the idea that the body is ultimately able to self-heal if impediments such as subluxations are removed leads to a type of thinking that sees vaccinations as external and ‘unnatural’ barriers to the bodies self-healing process. In this sense then, despite the belief having little if any scientific basis it may be expected, if not excused, within the context of a vitalistic paradigm. Despite understanding why these views may be held, the impact of failing vaccination uptake is far from trivial with a growing number of outbreaks of previously uncommon viral infections postulated to being associated with dropping vaccine adherence [27] impacted by antivax campaigns, particularly false claims around the aetiology of autism [28, 29]. Whilst there may be a legitimate debate around the timing and cost effectiveness of some vaccinations, there is overwhelming scientific support for high benefit versus harm [30] and no scientific support for the idea that MMR causes autism [31]. In the face of this scientific consensus, chiropractors views concerning supposed danger of vaccinations are unfounded and if passed on to patients, run counter to the expected behaviour of health care professionals.

There are clear limitations to this study. McGregor et al. [2] used a randomisation process to generate a smaller target sample, which increased their response rate and likely generalisability. In comparison, the inability to use this approach in this study and the relatively low response rate has the potential to compromise the generalisability of these results. Moreover, the potential unknown differences in dissemination to various subgroups through non participation by some associations may have also biased the responses. Lastly, erroneous indication of country of practice may have led to practitioners outside of Europe being included in the survey although we consider this to be unlikely to be large numbers.

However, despite the differences in response rate and sample size, results in this questionnaire were highly consistent with those obtained by other authors with 20.1% of the respondents identifying with the unorthodox views compared to the 18.8% in the original Canadian survey [2]. Additionally, other studies [32, 33] found a similar division when questioning chiropractors in the United States and chiropractors worldwide, where respectively 19.3 and 17.4% of practitioners aligned with the view suggesting the chiropractic subluxation as an encumbrance to the expression of health. Given the similarity to findings within surveys using methodology more likely to be generalizable to their populations, unless highly coincidental, may strengthen the possibility of representatitiveness in this study. In this context we also modified the original questions of the original survey and this may have reduced the validity of the result. However, again, the association between similar unacceptable practices and the unorthodox subgrouping found here and in McGregors’ study [2] suggests that the predictive validity of our survey was likely maintained.

Regarding the inclusion/exclusion of countries included in part of the analysis, the authors made an arbitrary cut off of at least a hundred chiropractors within the country according to the latest ECU registry (personal communication, 12 June 2017) and an individual response rate per country of 10% or higher. This does not allow us to make robust conclusions regarding the other counties or the true state of proportions of these groups within a country, and these results must be treated with caution.

## Conclusions

This study showed that around one fifth of European chiropractors completing this survey identified themselves with an a priori defined unorthodox description of chiropractic care. The data also showed a number of key predictive practice behaviours statistically associated with this unorthodox group, including higher use of x-rays, higher patient visits, absence of differential diagnostic approaches and less favourable views regarding the benefits of vaccination. Being the first of its kind in Europe, this study provides a unique description of beliefs and behaviours associated with unorthodox descriptions of chiropractic practice and may ultimately assist in understanding how such stances effect clinical behaviour.

The future and identity of the profession remains under constant, even exigent debate. Some chiropractors aspire to a more integrated approach into mainstream health care, whereas others wish chiropractic to remain sovereign, even antithetic to mainstream healthcare. Unfortunately, the chiropractic profession continues to engage in an internal battle around orthodox and unorthodox paradigms which has and continues to impede progression towards inclusion in a modern multidisciplinary health care setting and social and cultural legitimacy. Perhaps it is up to emerging generations of chiropractic students to find a solution to this schism [34] if the profession is to flourish and avoid an increasing and perhaps fatal marginalisation.
